# Healthy Older Observers Show Equivalent Perceptual-Cognitive Training Benefits to Young Adults for Multiple Object Tracking

**DOI:** 10.3389/fpsyg.2013.00323

**Published:** 2013-06-06

**Authors:** Isabelle Legault, Rémy Allard, Jocelyn Faubert

**Affiliations:** Visual Psychophysics and Perception Laboratory, School of Optometry, Université de Montréal, Montreal, QC, Canada

**Keywords:** aging, perceptual-cognitive training, learning, 3D-MOT, attention

## Abstract

The capacity to process complex dynamic scenes is of critical importance in real life. For instance, traveling through a crowd while avoiding collisions and maintaining orientation and good motor control requires fluent and continuous perceptual-cognitive processing. It is well documented that effects of healthy aging can influence perceptual-cognitive processes (Faubert, 2002) and that the efficiency of such processes can improve with training even for older adults (Richards et al., 2006). Here we assess the capacity of older participants to improve their tracking speed thresholds in a dynamic, virtual reality environment. Results show that this capacity is significantly affected by healthy aging but that perceptual-cognitive training can significantly reduce age-related effects in older individuals, who show an identical learning function to younger healthy adults. Data support the notion that learning in healthy older persons is maintained for processing complex dynamic scenes.

## Introduction

In our daily activities, we constantly interact with our environment. This environment is dynamic and requires the integration of various objects, motions, speeds, locations etc. There is ample evidence that the healthy aging process affects visual perceptual processing. Age-related deficits are particularly noticeable when the cognitive processes involved in the integration of information are more complex and require simultaneous assimilation of many aspects of the environment (Faubert, 2002). For example, in order to avoid collisions and be efficient in our displacements while driving, walking down a busy street, or through a crowded shopping center, we must process rapid movement and react quickly. In addition, our attention is distributed to encompass numerous elements simultaneously; for example, crossing the street requires an evaluation of traffic and pedestrian movement dynamics while maintaining a navigational goal. To conduct this efficiently, all the information available in our visual field must be integrated. Researchers report that older adults have difficulty with dividing their attention between a central stimulus and another stimulus simultaneously presented in the peripheral visual field (Ball et al., 1988; Richards et al., 2006) along with processing of complex motion (Habak and Faubert, 2000; Bennett et al., 2007; Tang and Zhou, 2009). This is generally consistent with verbal reports by older individuals that getting older has repercussions on their daily life (Kosnik et al., 1988).

A perceptual-cognitive task of particular relevance for exploring multifocal attention and complex motion information is multiple object tracking (MOT). MOT is a task where the observer is required to simultaneously track multiple moving items among many. The ability of the observer is typically evaluated by the number of elements that can be tracked successfully (Pylyshyn, 1989), and performance decreases with the number of targets (Pylyshyn and Storm, 1988; Yantis, 1992). Furthermore, a recent study has demonstrated that speed is a critical factor that is independent of other events in the scene, such as collisions, number of distractors, and distance between target and distractor (Feria, 2013). Pylyshyn and Storm (1988) proposed the FINST model to explain how people track items. Their model, based on primitive vision mechanisms, suggests that the visual system assigns preattentive indices to each element that works independently (Pylyshyn, 1994). On the other hand, Yantis (1992) proposed that targets are grouped together to form a higher-order perceptual representation or a virtual polygon, which requires a single attentional channel; this grouping is maintained during motion and facilitates tracking. However, more recent work has proposed that the visual system deploys a multifocal attention mechanism to keep track of the moving items (Cavanagh and Alvarez, 2005).

Regardless of the models to explain how people track multiple objects, older observers are less efficient at tracking multiple objects (Trick et al., 2005; Sekuler et al., 2008). Trick et al. (2005) have demonstrated that performance of older observers declines with an increasing number of objects being tracked. In their conditions, the maximum number of items that older adults could track was around three, whereas younger adults could track up to four. They suggested an age-related deficit in either the ability to report item position or to follow the position of multiple items. On the other hand, Sekuler et al. (2008), have suggested that age-related deficits are associated with tracking time, where longer tracking time and higher displacement speed reduce performance in older observers.

A number of questions remain, however, in regards to the perceptual-cognitive abilities of older observers. Some obvious questions are whether older observers’ capacity and learning rate differ from that of younger adults. Previous studies on divided attention with the useful field of view (UFOV) have shown that older adults could benefit from training (Ball et al., 2002; Richards et al., 2006; Edwards et al., 2009). However, the UFOV task, although useful for assessing dual attention throughout the visual field, does not directly assess dynamic scene processing. Faubert and Sidebottom (2012) demonstrated that remarkable improvements on the 3D-MOT task can be achieved with younger adults in a relatively short time. As argued by Faubert and Sidebottom, the potential for transferability of training on a perceptual-cognitive task to real-life situations like sports, navigation and driving, depends on a number of factors including visual field size, the necessity for tracking multiple dynamic objects, the presence or absence of stereoscopy (Viswanathan and Mingolla, 2002; Tinjust et al., 2008), and the use of speed. In a recent study we showed the relevance of 3D-MOT training by demonstrating that it can effectively transfer to a socially relevant percept such as biological motion, under conditions that are critical for collision avoidance (Legault and Faubert, 2012). We had previously demonstrated that the distance of a walker in virtual space impacted older observers’ abilities to determine walking direction (Legault et al., 2012). In the following study (Legault and Faubert, 2012) we tested three groups of older observers. One group was trained on the 3D-MOT task, another was trained on a visual task for the same duration, and a third was a control with no training. When the groups were subsequently assessed on the biological motion task, only the group trained on the 3D-MOT showed improvements.

The purpose of the present study was therefore to address these questions directly with two experiments. The goal of the first experiment was to determine if older observers have lower limits in their capacity to track multiple moving objects when compared to young adults (Trick et al., 2005). The second experiment addressed the training capacity of older observers compared to younger adults. If one assumes that aging reduces learning functions, then older observers should not progress at the same rate as younger observers. On the other hand if learning is maintained, then a similar progression rate as a function of training would be observed. Yet a third outcome is possible. If training can reverse age-related impairments, then older observers may show an even better learning rate.

## Experiment 1

In a MOT-type task where the dependent measure used was the maximum number of elements that could be tracked, Trick et al. (2005) suggested that older adults’ tracking ability was probably lower then young adults (three items in their conditions). Faubert and Sidebottom (2012) proposed that speed thresholds may be a more relevant and controllable measure of MOT demands than using the total number of items as a dependent measure. To readdress the proposed limitation of three items for older observers, Experiment 1 compared the speed thresholds of younger and older observers tracking three and four targets. If older observers are truly limited to three targets then we should not be able to obtain thresholds regardless of the speed of the elements. If on the other hand older observers can track four targets and the difference between tracking three and four targets is purely quantitative then we should find no group by target condition interactions. A group by target interaction would indicate both a capacity to track elements yet with greater relative difficulty for the older group.

### Methods

#### Participants

Ten younger adults (mean age 27 years old, range: 22–34 years old) and 10 older adults (mean age 66 years old, range: 61–74 years old) participated in this study. All participants were naïve to the purpose of the experiment. All subjects had normal or corrected-to-normal vision (6/6 or better) with normal stereoacuity as measured by the Frisby test (40 s of arc or better) (Sasieni, 1978; Frisby, 1980). Viewing was binocular. Younger adults had their most recent eye exam within the last year. Older adults were recruited at the school of optometry of Université de Montréal and had their most recent eye exam within the last 6 months. This eye exam included refraction, binocular vision evaluation, tonometry, visual field, retinal exam under pupil dilatation. Only subjects without ocular pathology or other abnormality were included in the study. Older observers completed the Mini-Mental State Examination, a screening measure for cognitive impairment and all scores were within the normal range (range, 26–30/30; subject mean was 28/30) (Crum et al., 1993). Therefore, they were all considered cognitively healthy. Ethical approval was obtained from the University’s ethics board.

#### Apparatus

The 3D-MOT task was assessed using a fully immersive virtual environment: the Cave Automatic Virtual Environment (CAVE) system. The CAVE was an 8 × 8 × 8 feet room that includes three canvas walls (one frontal and two laterals) and an epoxy floor that all serve as surfaces for image projection (Faubert and Allard, 2004). Four high-resolution projectors were synchronized and the image was updated in real-time to maintain the true viewing perspective of the observer (no false parallax). A magnetic motion tracker system (Flock-of-Birds) was used to measure head position, which was used to correct for the viewing perspective of the observers in real-time. The CAVE was under the computer control of an SGI ONYX 3200 (two Infinite Reality 2 graphics cards) generating a stereoscopic environment. The stereoscopy was generated with Crystal Eyes 2 active shutter glasses synchronized at 96 Hz (48 Hz per eye).

#### Stimuli and procedure

**Figure 1:**
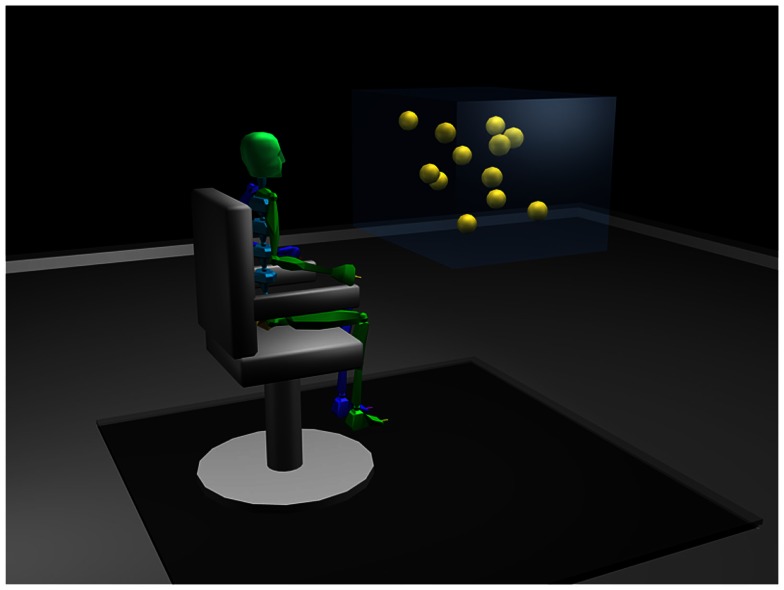
**Illustration of the experimental 3D-MOT set-up in the CAVE environment**. The walls of the virtual cube are shown here for illustration purposes. They were not visible in the actual set-up.

Before testing, participants were familiarized with the virtual environment and the stimulus. They were then asked to wear the stereoscopic goggles, which allowed them to perceive the 3D characteristics of the environment. Each participant sat at 177 cm from the central wall of the CAVE with eye height set at 160 cm from the ground. They were asked to stare at the fixation point, located straight ahead. Stimuli consisted of nine spheres projected into a virtual cube having transparent virtual light blue walls. The anterior side of the cube measured 42 ° of visual angle and was seen at 57 cm. The spheres followed a linear trajectory in the 3D virtual space but were bouncing on one another and on the walls when collisions occurred (see Figure 1).

**Figure 2:**
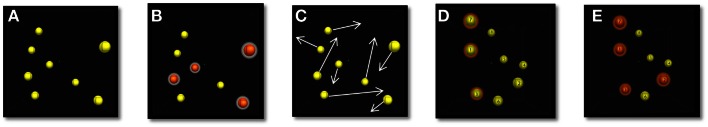
**Illustration of the five critical phases: (A) presentation of randomly positioned spheres in a virtual volumetric space, (B) identification of the spheres to track during trial, (C) removal of identification and movement of all spheres with dynamic interactions, (D) observer’s response by identifying the spheres, (E) feedback was given to the observer**.

We used the 3D-MOT speed threshold protocol (Faubert and Sidebottom, 2012). There were two conditions: three or four targets to track. Each block (i.e., staircase) lasted about 10 min. In one session, participants ran three staircases per condition (three or four spheres), for a total of 60 min. Each trial had five phases (see Figure 2). *Phase 1, presentation phase*: nine yellow static balls were presented for 2.5 s in random positions. *Phase 2, indexation phase*: three or four spheres turned red for 2 s for target identification, before turning back to yellow. *Phase 3, tracking phase*: all spheres started moving in random directions at a speed defined by the staircase procedure (see below) and bounced off of one another and off the virtual walls for 10 s. *Phase 4, identification phase*: the spheres stopped moving and each had a unique identification label from 1 to 9. The participants’ task was to report, verbally, the 3 (or 4) numbers corresponding to the three (or four) target spheres that had been indexed. *Phase 5, feedback phase*: feedback was given, showing the observer the spheres that were initially indexed. Speed thresholds were then evaluated using a one up one down staircase procedure (Levitt, 1971) that is, after a correct response, ball speed displacement was increased by 0.05 log units and decreased by the same proportion after each incorrect response, resulting in a threshold criterion of 50%. The staircase was interrupted after eight inversions and the threshold was estimated by the geometric mean speed of the last four inversions. The initial virtual speed was fixed at 3.75 cm/s. To obtain a correct answer, participants had to correctly report all the targets.

### Result and discussion

From the 10 older participants, only nine were included in the analysis. One could not perform the task even for three targets at very low speeds (e.g., 1 cm/s). This participant was considered as an outlier since he was the only one from 30 (including 20 in Experiment 2) that could not complete the task. A 2 × 2 split-plot ANOVA on log speed thresholds revealed a significant Age (between variable) × Number of targets (3 or 4, within variable) interaction, *F*(1, 17) = 6.45, *p* = 0.021. Subsequently, paired *t*-test showed significant differences for number of targets; younger adults: *t*(1, 9) = 3.480, *p* = 0.007, older adults: *t*(1, 8) = 8.204, *p* < 0.001.

**Figure 3:**
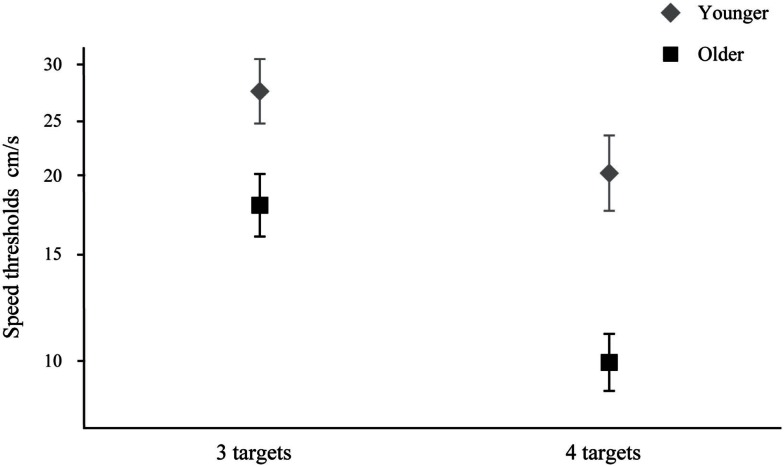
**Ten younger and nine older adults speed thresholds (±SEM) for three and four targets**.

Results show that older adults can successfully follow three and four targets, but at significantly lower speeds (Figure 3). Healthy aging clearly reduces the ability to track many objects. Performance for younger adults with four targets is similar that with three targets for older adults. Furthermore, as shown by a significant interaction, performance decline between three and four spheres is more pronounced for older adults, suggesting that they have difficulty managing more information and require more time to process it. Our results show that older observers are not limited to tracking three targets. However, we do show greater relative difficulty for tracking four targets in this population as evidenced by the significant interaction, which partially supports the conclusion of the Trick and colleague study that healthy older observers have more difficulty tracking four targets.

## Experiment 2

In the second experiment we trained younger and older adults during five consecutive weeks on the 3D-MOT task to determine the performance progress for younger and older adults. Here we wanted to determine which of the three possible outcomes prevails for 3D-MOT: either (1) they have a slower learning rate than younger adults; (2) they still have equivalent learning functions; or (3) training can reverse some age-related impairment, in which case training should reduce the performance difference between both age groups.

### Methods

#### Participants

Two new experimental groups participated in this experiment. One group was composed of 20 young observers (mean age 24 years old, range: 18–35 years old) and another consisted of 20 older observers (mean age 67 years old, range: 64–73 years old). All observers came to the lab once a week for five consecutive weeks. The same inclusion and exclusion criteria were used as for Experiment 1. Again, the older observers’ scores on the Mini-Mental State Examination were all within the normal range (range, 28–30/30; subject mean was 29/30) (Crum et al., 1993). Therefore, they were all considered cognitively healthy. All participants completed a short survey on video game habits. The survey questions are listed below.

Name of the game
Electronic game device
Number of hours per session
Number of sessions per month

#### Stimuli and procedure

The same set-up, stimuli and procedure as in Experiment 1 were used. We used the three-target protocol and for every session, participants ran three blocks (three thresholds) for a total of a 30-min session per week.

### Results and discussion

**Figure 4:**
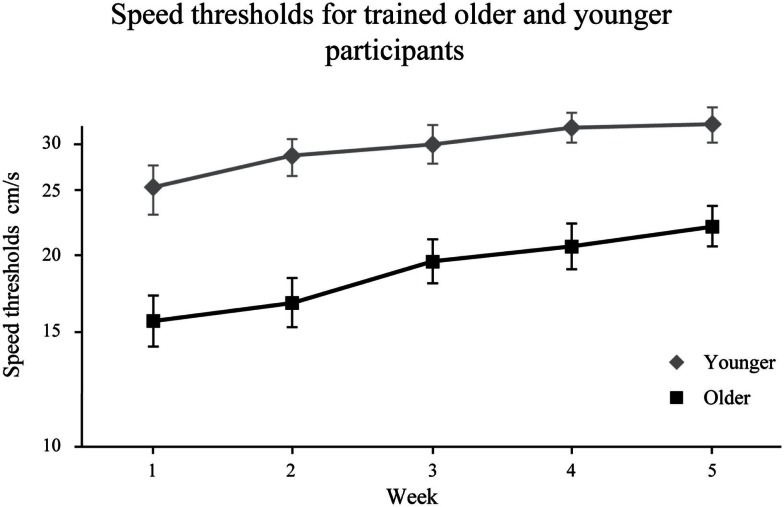
**Twenty younger and 20 older adults speed thresholds (±SEM) for five consecutive weeks**.

A split-plot ANOVA on log speed thresholds comparing post-training condition (week 5) versus the initial scores (week 1) revealed a significant group effect, *F*(1, 41) = 18.250, *p* < 0.001 and a significant training effect *F*(1, 41) = 65.747, *p* < 0.001. Specifically, younger adults obtained higher thresholds compared to older adults and thresholds increased with testing sessions (Figure 4). However, we did not obtain a significant Age × Training interaction, *F*(1, 41) = 2.615, *p* = 0.114, which reveals that both groups obtained similar benefit from training. There is a similar progression for both groups. Figure 4 shows that trained older observers obtained thresholds that were similar to those of untrained younger adults (week 1) [*t*(1, 19) = 0.495, *p* = 0.626].

**Figure 5:**
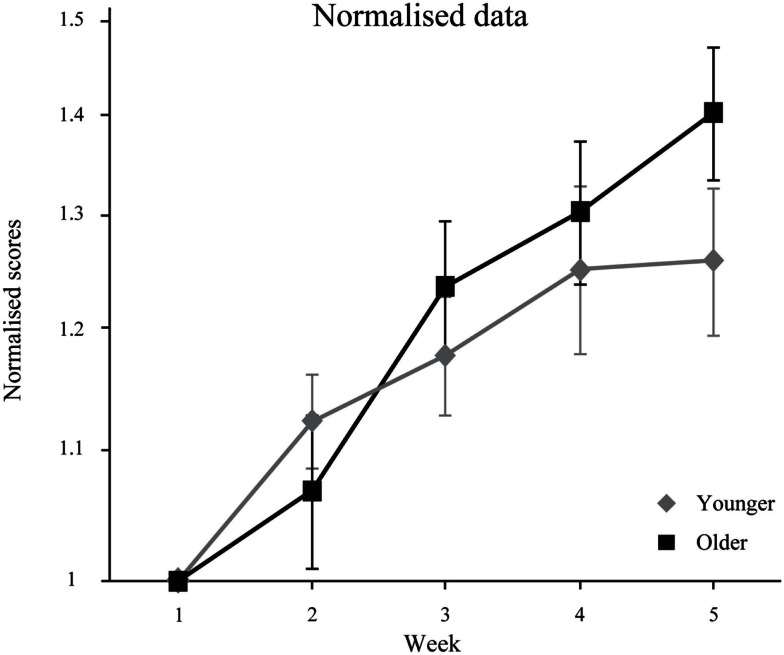
**Normalized speed thresholds (±SEM) for 20 younger and 20 older adults**. (Ratio of speed threshold relative to week 1).

Figure 5 represents normalized data relative to week-1 baseline scores and indicates that younger and older adults exhibit the same learning growth. However, at week 5, the improvement for older observers still appears on the rise, while younger observer group scores tend to level off. It is possible therefore, that improvement from training in the older group has not plateaued at 5 weeks. Interestingly, Richards et al. (2006) found that their older adults needed more practice to reach younger adult levels.

## General Discussion

In this paper, we assessed 3D-MOT for younger and older adults. In both experiments, we reported a significant age-related deficit, in which older adults obtained lower performance scores compared to younger adults. These results are generally consistent with previous MOT research showing reduced performance for older adults (Trick et al., 2005; Sekuler et al., 2008; Kennedy et al., 2009). Specifically, in Experiment 1, we showed that both younger and older adults obtained a significant reduction of their speed thresholds when their performances are compared for the three-target condition with the four-target condition. Trick et al. (2005), found that older adults could only track around three moving objects under their stimuli conditions. However, our results cannot be explained by the fact that observers only tracked three balls + one by chance. If this were the case, the fourth ball would be identified correctly only one time out of six (17% chance), and the staircase would, on average, decrease ball speed. However, all thresholds obtained were higher than the initial starting staircase value of 3.75 cm/s. For the staircase to increase speed after the initial start value, the probability of identifying the correct four balls had to be higher than 50%, so observers must have followed all four balls. Older adults can track four items but they need a reduction of target movement speed to achieve this. Our results are generally consistent with previous studies showing that target quantity (Pylyshyn and Storm, 1988; Yantis, 1992; Alvarez and Franconeri, 2007) and object speed (Alvarez and Franconeri, 2007; Sekuler et al., 2008; Faubert and Sidebottom, 2012), are critical factors influencing performance. Trick et al. (2005) suggested that the difficulty for older participants to track four balls could be related to maintaining ball representation in working memory, until observers could report the targets. If an age-related effect on speed threshold was due to the ability of older observers to report four targets, then performance with slow speeds should have also been affected. However, at the slowest speed with four targets (i.e., staircase initial value of 3.75 cm/s) the percentage of correct trial was similar for older (89%) and young (90%) observers. Therefore, the difference between groups is unlikely related to working memory capacity alone. Furthermore, collisions cannot totally explain the differences observed between three and four targets. The proportion of collisions that occurs between a target and a non-target is, on average, 0.5357 (2 × 3 × 5/8 × 7) and 0.5714 (2 × 4 × 4/8 × 7) when tracking three and four balls out of eight, respectively. So everything else being equal, there will be only 1.0666 times more collisions between targets and non-targets when tracking four instead of three targets. So, if we measure observer’s performance in relation with the number of collisions, we still have significant difference in both groups between three and four balls conditions [younger: *t*(1, 9) = −1.099, *p* = 0.024), older: *t*(1, 8) = −7.275, *p* < 0.001]. Consequently, the number of collisions cannot explain, by itself, the reduced speed thresholds when tracking four rather than three targets. This is consistent with Feria’s (2013) conclusion that MOT performance cannot only be explained by the number of collisions. Nevertheless, 3D-MOT is a complex task that can be sensitive to many factors related to dividing attention, tracking, stereopsis, collisions, and occlusions. Thus, further experiments will be required to identify which functions may be affected by aging.

In Experiment 2, results show that both younger and older adults obtained similar training gains from 3D-MOT. Their performance after 5 weeks of training was significantly improved; participants processed the 3D-MOT task successfully at faster speeds. Overall, younger adults show better performance than older adults but the lack of significant interaction shows similar training gains between groups. Figure 5, clearly shows this trend. Our findings are consistent with previous work showing improvement of perceptual processing with training (Kramer et al., 1995; Richards et al., 2006; Andersen et al., 2010). Specifically, tasks become more automatic with training, reducing attentional costs (Ma et al., 2010).

As expected, our results show that younger adults obtained better performance than older adults. An element that can contribute to higher performance in younger adults is their previous exposure to certain stimuli as found in video games (Sekuler et al., 2008). Younger adults tend to be more exposed to video games, internet, television, etc. Some studies have shown that expertise can increase the ability to track multiple items (Green and Bavelier, 2006). However, we conducted a video game habit survey for each of our participants and video game experience cannot explain the difference between younger and older adults, because our participants did not play at any video games more than once a week except for one gamer (based on Green and Bavelier, 2006 criteria) and his MOT speed thresholds were not substantially different from the others. On the other hand, it could be assumed that older adults are less exposed to complex dynamic scenes in their everyday activities. It is possible that older observers engage less in certain activities because they have difficulty processing complex visual scenes.

In the present study, our 3D-MOT task was conducted in a virtual reality environment soliciting information integration across a large visual field. Consistent with previous research using UFOV (Richards et al., 2006), we observed that both younger and older adults can be trained and their performance improved, on an attention related task presented over a wide visual field. Richards et al. (2006), trained younger and older adults with an UFOV task, where central and peripheral vision and dual-tasking was required (see Figure 4 in Richards et al., 2006). After enough practice, older adults reduced the attentional cost to levels similar to those of younger adults, and both groups achieved performance without any divided-attention deficit, something we could not achieve with the 3D-MOT under our conditions. There are a number of differences between our MOT task and the Richards et al. (2006)’s UFOV task: our stimuli were dynamic, and the visual field size was twice that used by Richards et al. (2006). These differences may impact transferability to real-life situations. In previous studies, data show that older adults needed more training sessions to reach younger adult performance levels (Salthouse, 1990; Kramer and Willis, 2002; Richards et al., 2006). Our data (Figure 5) indicate that older adults’ progression looks like it had not yet peaked, and there may still be room for improvement. More training sessions would be needed to determine if results obtained at week 5 reflect peak learning levels. To address this issue, we have conducted a pilot study in which we tested the same conditions as in Experiment 2, on eight young and eight older subjects for 10 instead of 5 sessions. The results did not conclusively show any closing of the gap between older and younger observers. From week 5 to week 10, younger adults maintained higher speed thresholds; however the learning progression was identical for both groups. The ANOVA shows a significant group effect [*F*(1, 41) = 22.871, *p* < 0.001] but no significant interaction [*F*(4, 164) = 1.967, *p* = 0.102)]. Furthermore, we have calculated the linear [*F*(1, 41) = 3.342, *p* = 0.075] and the quadratic [*F*(1, 41) = 0.896, *p* = 0.349] trends, and results showed similar learning rates in both groups (i.e., no learning × group interaction). Given this lack of interaction one could propose that, in addition to learning, the older group is also gaining familiarity to the computer. We believe this is not the case for several reasons. First of all, we have previously shown that the same basic technique is quite sensitive to different levels of learning as demonstrated by a recent paper comparing pro-athletes, elite amateurs and university students (Faubert, 2013). Given the huge difference observed in the learning curves in these three categories for the same number of total thresholds reported here in the first phase (15 thresholds), if there was a significant difference between learning of the control group here and the older observers, we should have been quite sensitive to it early on. Secondly, it is important to note that the observers in this task are always passive, i.e., there is no interaction with the computer system and the subjects only identify the target spheres after the trial is over and therefore there is no motor control or dual-tasking in this regard. It is a passive viewing task and the only thing the subjects are learning is how to track the targets at increasing speeds. Thirdly, we have previously shown that older subjects (same age group as the present study) that trained in the same environment (CAVE) but on spatial discrimination task, showed very little learning and zero transfer to another dynamic task such as biological motion perception (Legault and Faubert, 2012). This was not the case for the group that trained on the 3D-MOT task that showed transfer to biological motion perception task at 4 m even if they had never seen the biological motion task before (see Legault and Faubert, 2012). Taking these points together, we feel it is highly unlikely that habituation to the computer set-up would explain our results with the older observers. Note that researchers have shown, using other research paradigms, transfer in everyday tasks (Tucker-Drob, 2011). There is further evidence that older adults with expertise can have high performing levels on real-world tasks (Nunes and Kramer, 2009). It would therefore be quite interesting to study the combined impact of expertise and training with the 3D-MOT task in future studies.

Our findings can be summed into two main points. We show that perceptual-cognitive training for a complex dynamic multifocal attention motion task, such as 3D-MOT, can be trained and, more importantly, that the training benefit for older observers is of the same magnitude as that for younger healthy adults. Consequently, it would certainly be worthwhile to conduct future studies on the transferability of this training to real-life tasks, such as driving or other socially relevant tasks.

## Conflict of Interest Statement

Isabelle Legault and Rémy Allard declare that the research was conducted in the absence of any commercial or financial relationships that could be construed as a potential conflict of interest. Jocelyn Faubert is the Chief Science Officer of CogniSens Inc., a company that has signed a licensing agreement for four technologies from the Université de Montréal and produces the NeuroTracker ™system, the commercial version of the 3D-MOT speed task.
